# Efficient Route for the Preparation of Composite Resin Incorporating Silver Nanoparticles with Enhanced Antibacterial Properties

**DOI:** 10.3390/nano12030471

**Published:** 2022-01-29

**Authors:** Drake Beery, Mohammad Abdul Mottaleb, Mohammed J. Meziani, James Campbell, Isabella Pires Miranda, Michael Bellamy

**Affiliations:** 1 Department of Natural Sciences, Northwest Missouri State University, 800 University Drive, Maryville, MO 64468, USA; S513946@mail.nwmissouri.edu (D.B.); jhcamp@nwmissouri.edu (J.C.); bswb_spring@iie.org (I.P.M.); bellamy@nwmissouri.edu (M.B.); 2 Center for Innovation and Entrepreneurship, Northwest Missouri State University, 800 University Drive, Maryville, MO 64468, USA; 3 Institute of Drug & Biotherapeutic Innovation (IDBI), Office of the Vice President for Research Doisy Research Center (DRC), Saint Louis University, 1100 South Grand Blvd, Saint Louis, MO 63104, USA; mabdul.mottaleb@slu.edu

**Keywords:** silver nanoparticles, resin beads, nanocomposites, antibacterial properties

## Abstract

An efficient and facile route for the immobilization of silver (Ag) nanoparticles (NPs) in anion exchange resin beads with different silver loading is proposed. In this method, BH_4_^−^ ions were first introduced into chloride-form resin through an ion exchange process with Cl^−^ ions, followed by in-situ chemical reduction of Ag^+^ ions at the surface of the resin to form metallic Ag nanoparticles. Morphology and structure of the resulting Ag-resin nanocomposites were characterized by X-ray diffraction (XRD), scanning electron microscope (SEM), energy dispersive spectroscopy (EDS), Fourier transform infra-red (FTIR), inductively coupled plasma-optical emission spectrometry (ICP-OES), and thermogravimetry analysis (TGA). The results confirmed the presence of smaller diameter Ag NPs incorporated into the resin beads having an average diameter on the order of 10 nm with a few Ag NP clusters of 20–100 nm. The nanoparticles were homogeneously distributed throughout the resin. There were no dramatic increases in average particle sizes even at very high Ag loadings. The resin retained its structure and stability, allowing higher stability of immobilized AgNPs than the colloidal ones. The Ag-loaded resins made with 50 mM AgNO_3_ were tested for antibacterial activity in vitro against *Escherichia coli* (*E. coli*) as a model microbial contaminant in water. Results showed greater than 99% bacterial inhibition within 3 h of exposure. The resin form offers greater ease of handling, long-term storage at room temperature, reusability in repeated reactions, and reduces the risk of environmental contamination.

## 1. Introduction

In recent years, the immobilization and storage of metal nanoparticles in a solid resin matrix has offered enormous possibilities for the design of functional materials with significant technological applications. These nanocomposites have been exploited for the development of antibacterial materials [1,2,3,4], catalysts [5,6,7,8], water treatment [9,10,11], and surface-enhanced Raman spectroscopy substrates [8,12,13]. Compared with other systems, the employment of ion-exchange resins offers several advantages for the assembly of nanoparticles, including superior chemical and thermal stability without agglomeration, ability to control the purity and homogeneity of the final material, availability at low cost, no health risk and environmental contamination, ease of handling and recycling, and facile integration into existing technologies. Because of these unique features, various types of ion-exchange resins have been explored in the immobilization of various metal nanoparticles, leading to their successful applications [1,2,3,4,5,6,7,8,13,14,15,16]. The synthetic procedures usually involved immobilizing the metal ion onto the resin by ion exchange using an appropriate soluble metal precursor, followed by metal reduction [7,8]. Ag has particularly attracted considerable attention among all the metals due to its unique optical, catalytic, and antibacterial properties [17,18,19,20]. Ag has many advantages, such as low toxicity, good biocompatibility with human cells [20,21,22], and potent long-term antibacterial activity, due to sustained ion release and efficient penetration through cell membranes [23]. Their smaller size increases the surface area and enhances the chemical activity, providing potent antibacterial effects at lower concentrations [17,20,24].

As a result, preparation of mono-disperse and uniform Ag nanoparticles coated resins has been of special interest, and so far, only very few methods have been reported. For example, Nath et al. [12] reported the synthesis of an ultrathin Ag nanoshell (~50 nm) on polystyrene beads using [Ag(NH_3_)_2_]^+^ as a metal precursor for cation-exchange resins. The resin beads loaded with [Ag(NH_3_)_2_]^+^ were heated to form Ag_2_O and then reduced with sodium borohydride solution. Similarly, Ag coated resin beads were prepared by trapping silver precursor ions [AgCl_2_]^−^ in the anion exchange resin beads through exchange with Cl^−^ ions and then reducing them with sodium borohydride [25]. The as-prepared reddish-black beads showed the presence of spherical Ag NPs of ~30 nm diameter in the resin matrix. In another report, Ag oxide NPs were also stored in anion exchange resin beads by first activating the resins with OH^−^ and NO_3_^−^ ions and then exchanging them with a colloidal solution containing Ag NPs, which have an overall negative charge on their surface [26].

Despite these advances, most reported approaches still have some restrictions or shortcomings that limit their extensive application. These include the use of complex precursors for exchange, often requiring more than one production step, and the use of pre-fabricated nanoparticles mixed with resin, which tend to cause agglomeration, inhomogeneous distribution, and poor adaptability to other metals.

In the present article, we report a facile and efficient approach for preparing Ag NPs (diameters mostly on the order of 10 nm) coated anion exchange resin beads with different silver loading. In this approach, BH_4_^−^ ions were first introduced into the resin through an ion exchange process with Cl^−^ ions, followed by wet chemical reduction of Ag^+^ ions with BH_4_^−^ ions at the surface of the resin to form metallic Ag nanoparticles. Morphology and structure of the resulting Ag-resin nanocomposites were characterized by XRD, FTIR, SEM, EDS, and TGA. While hosted in the cavities, the Ag nanoparticles were found to be mostly stable in ambient air. Finally, the resin-bound Ag nanocomposites have been successfully exploited for their antimicrobial activity in vitro against *E. coli* as a model microbial contaminant in water.

## 2. Materials and Methods

### 2.1. Materials

Purolite^®^ A530E in the chloride form (Purolite is a leading manufacturer of ion exchange resins) was supplied by Lenntech. It is a macroporous strong base anion with triethylammonium exchange sites and the polystyrene backbone crosslinked with divinyl-benzene. According to the supplier, the exchange capacity of this resin is 0.6 eq/L, and the water retention capacity ranges between 50 and 57%. The resin has good mechanical strength and excellent osmotic and thermal shock resistance. It can withstand temperatures from −40 °C to 100 °C [27]. The resin was specifically designed to remove perchlorate (ClO_4_^−^) and pertechnetate (TcO_4_^−^) from contaminated groundwater. Water was deionized to 18.2 MΩ cm using a Millipore Milli Q UVPlus (MilliporeSigma, Burlington, MA, USA) water purification system. Silver nitrate (AgNO_3_) was used as received from Aldrich. Sodium borohydride (NaBH_4_) was used as received from ACROS. Nitric acid (HNO_3_) was obtained from Fisher. Nutrient broth and agar were purchased from Difco and Fluka, respectively. The microbial strain used for the inactivation experiments was *Escherichia coli* B (Microbiologics KWIK-STIK derived from ATCC 8739; St. Cloud, MN, USA).

### 2.2. Synthesis of Ag NPs Coated Anion Exchange Resin Beads

A two-step procedure synthesized the Ag nanoparticles embedded in resin. The first step involved the adsorption of BH_4_^−^ ions on the chloride-form resin through an ion exchange process with Cl^−^ ions. The second step was the reduction of Ag^+^ ions at the surface of the resin to form metallic Ag nanoparticles. For the first step, 2 g of Purolite^®^ A530E resin were mixed with 20 mL fresh ice-cold aqueous solution of 0.5 M NaBH_4_ and stirred continuously for 2 h to allow anion exchange to occur. The resulting resin was washed with distilled water thoroughly until free from excess sodium borohydride. The borohydride exchange resin was then dried in a vacuum at 65 °C for two hours. In the second step, 20 mL aqueous AgNO_3_ with known concentration was added dropwise to the dry resin containing BH_4_^−^ in an ice bath while stirring. The film surface was washed thoroughly with deionized water to remove any residual silver salt and then dried in a vacuum for two hours. The obtained resin-bound Ag NPs were stable in air.

### 2.3. Ag-Loaded Resins Characterization

Scanning electron microscopy (SEM) imaging was performed on a JOEL Field Emission Scanning Electron Microscope Model JSM7600F (FE-SEM, JEOL, Tokyo, Japan) for each sample to determine the size, distribution, and morphology of Ag-loaded resin. X-ray diffraction (XRD) was used to determine the crystal phase of the Ag nanoparticles incorporated into resin. The resin embedded with Ag nanoparticles was crushed via grinding in a mortar to form powdery materials. The XRD measurements were recorded on a Rigaku MiniFlex II Desktop X-ray diffractometer using CuKα radiation with 1.5416 Å wavelengths. The diffractometer was set to analyze the samples at angles between 5 and 80 degrees (2θ) at the rate of 1 degree of angle increase per minute. The crystallite size of the Ag nanoparticles loaded in resin were determined from X-ray line broadening using the Debye–Scherrer equation D = K λ/(β cos θ), where D = crystallite size, A (Angstroms), K = crystallite-shape factor = 0.9, λ = X-ray wavelength, 1.5416 Å for CuKα, θ = observed peak angle, degree, β = X-ray diffraction broadening in radian [28].

Surface functionality was evaluated by Fourier transform infrared (FTIR) spectroscopy using a Perkin Elmer Spectrum One spectrometer equipped with a ZnSe Attenuated Total Reflectance accessory. Each spectrum was obtained with an average of 200 scans at 4 cm^−1^ resolution. The interferograms were apodized with a cosine apodization function.

The Ag contents in the resin were estimated by analyzing the thermal decomposition of pure resin and Ag-loaded resins using a thermogravimetric analyzer (TGA4000, Perkin Elmer, Oak Brook, IL, USA. Approximately 5 mg of sample was decomposed under a nitrogen flow at 20 mL/min with a constant heating rate of 10 °C/min up to a maximum of 900 °C.

The Ag contents in the saturated resin and that released from the Ag/resin beads after 3 h shaking in nutrient broth were quantified by optical emission spectrometry with inductively coupled plasma (ICP-OES, Varian 710-ES, Victoria, Australia). Before the ICP analysis, the samples were digested in concentrated nitric acid (HNO_3_, Synth) for 24 h and suspended in ultrapure water, which facilitates retrieval of all AgNPs from the resin and converts them into ionic form. After required dilution, the silver content was automatically determined based on the calibration curve generated using a Sigma Aldrich ICP silver element standard solution.

### 2.4. Bactericidal Activity and Statistical Analysis

Bacteriocidal properties of Ag-loaded resin beads were determined using standard viable counts of *E. coli* B grown in nutrient broth or on nutrient agar (no NaCl added) for all manipulations. Cells were grown overnight and diluted to 10^−4^ in sterile broth. Dilutions in sterile saline (0.85% NaCl) were spread plated to determine initial cell densities. Aliquots of 250 μL were added to wells of a 96-well-microtiter plate containing only cells, cells with a single unloaded resin bead, or cells with a single Ag-loaded resin bead. Cells were incubated at 37 °C with shaking (150 rpm). Three independent replicate wells of each treatment were sampled at 60, 120, and 180 min, at which time dilutions and spread plating (100 μL aliquots) were performed. Spread plates were incubated overnight at 37 °C and counted manually. Plates were considered countable when producing 30–300 colonies whenever possible. However, spread plates of undiluted samples (10^0^) from Ag-loaded resin beads dropped below this range at 120 and 180-min samplings. Therefore, observed plate counts were used.

A two-way analysis of variance (ANOVA) was used to test for significant effects of treatments (no bead, resin bead, resin + Ag bead) and exposure time (60, 120, 180 min) on bacterial viable counts. The ANOVA was calculated using the built-in “ANOVAN” function in Matlab (R2014a). Because the treatment-time interaction effect was found to be significant, post hoc t-tests were used to detect significant differences between treatments at each exposure time [29]. This was accomplished using the “TTestPairwise” function from a freely available statistics toolbox for Matlab [30]. Such multiple comparisons are known to inflate Type I error rates [29], so alpha values were Bonferroni-adjusted to control for multiple comparisons (α = 0.05/9 = 0.0056). In essence, only *p*-values less than 0.0056 are considered statistically significant.

## 3. Results and Discussion

**Figure 1:**
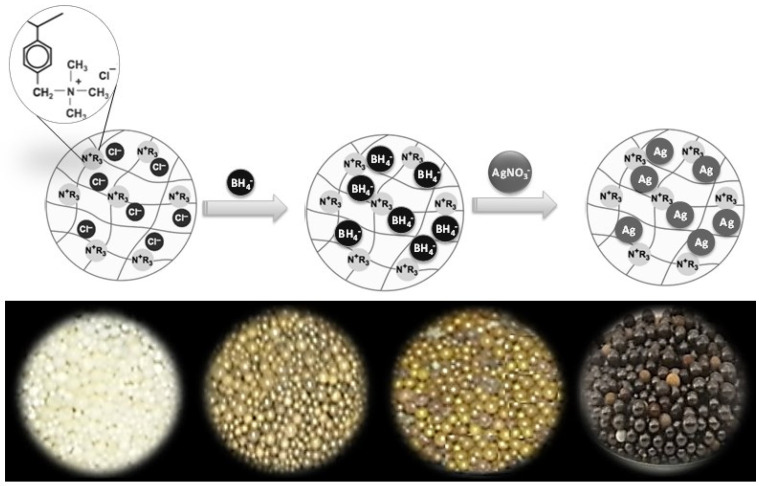
Schematic illustration of the preparation of a hybrid anion exchange resin impregnated with Ag NPs (**top**) and their visual appearance when prepared with increasing AgNO_3_ concentrations (**bottom**). The color of Ag-resin beads changed from yellow to yellow-brown and finally brown-black with increasing AgNO_3_ concentration.

The Purolite A530E resin used in this study is macroporous polystyrene crosslinked with divinylbenzene containing anion-exchanging quaternary ammonium groups as an integral part of the polymer lattice with an equivalent amount of Cl^−^ anions [27]. The material is supplied in the form of small beads having diameters of 0.5–1.0 mm and has a highly developed structure of pores (with a size of about 2 to 100 nm) on the surface and throughout the framework. The resin was used to support and immobilize Ag NPs into resin beads, as depicted in Figure 1. In this synthesis process, BH_4_^−^ ions were first introduced into the resin through an ion exchange process with Cl^−^ ions, followed by wet chemical reduction of Ag^+^ ions with BH_4_^−^ ions at the surface of the resin to form metallic Ag nanoparticles. This is different from previous reports in which either the metal ions were first immobilized by ion-exchange using soluble complex metal precursors before its reduction [12,25] or pre-fabricated nanoparticles mixed with resin [26] that often tend to cause agglomeration and inhomogeneous dispersion. As shown in Figure 1, the ion exchange of NPs also took place efficiently with other AgNO_3_ concentrations and showed a gradual change in color of the resin bead from yellow to yellow-brown and finally brown-black with increasing AgNO_3_ concentration.

**Figure 2:**
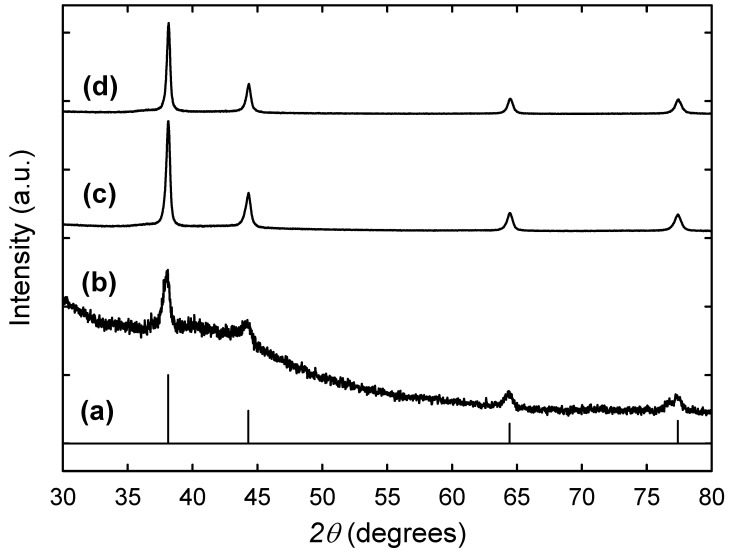
X-ray diffraction patterns of the Ag NPs embedded in resin beads prepared with increasing AgNO_3_ concentrations of 1 mM (**b**), 10 mM (**c**), and 50 mM (**d**) and compared with that of bulk fcc silver in the JCPDS database (**a**).

The synthesized Ag NPs in resin beads with increasing AgNO3 concentration were identified by their X-ray diffraction patterns. As shown in Figure 2, the peaks in all samples matched well with the standard for bulk Ag in the JCPDS database. The pure resin did not show any well-defined peaks, which indicated the amorphous nature of the sample. The observed Ag peaks at 2*θ* angle 37.7°, 44.0°, 64.2°, and 77.1° correspond to the four diffraction peaks (111), (200), (220) and (311) crystal planes, respectively, indicating the formation of face-centered-cubic (fcc) metallic Ag NPs within the resin beads. The XRD peaks were generally broad, consistent with the embedded Ag particles being nanoscale. By increasing the concentration of AgNO_3_, the peaks became well-defined and more intense due to the increase in the number of Ag NPs inside the resin structure. The peak broadening was used to estimate the average particle size in terms of the Debye–Scherrer equation indicated in Section 2.3. The average particle sizes thus estimated by this calculation were 11.3, 15.9, and 16.4 nm for the Ag nanoparticles in resin beads synthesized with AgNO_3_ concentrations of 1, 10, and 50 mM, respectively, indicating no significant change in the Ag particle size with increasing concentrations of AgNO_3_ in solution.

**Figure 3:**
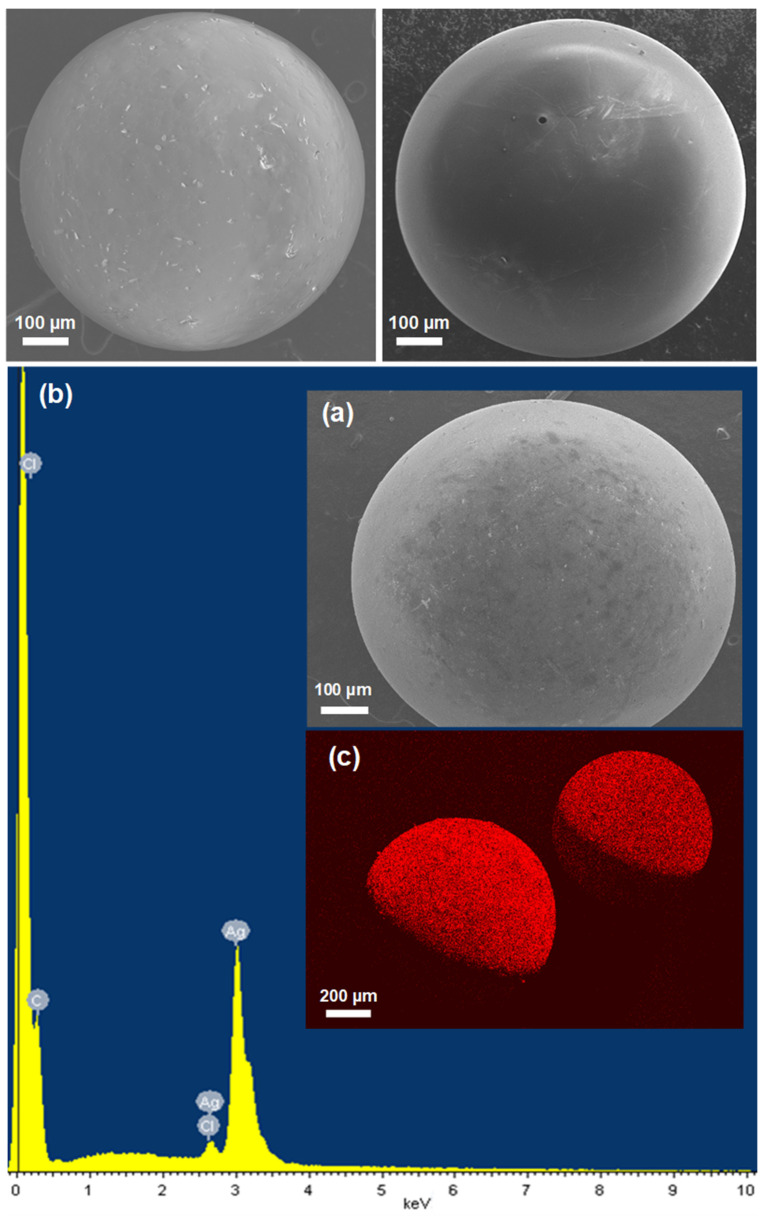
Top: Representative SEM images of the original unloaded resin beads. Bottom: Representative SEM image (**a**) and EDS spectrum (**b**) and mapping (**c**) for Ag element on the resin beads embedded with Ag NPs.

**Figure 4:**
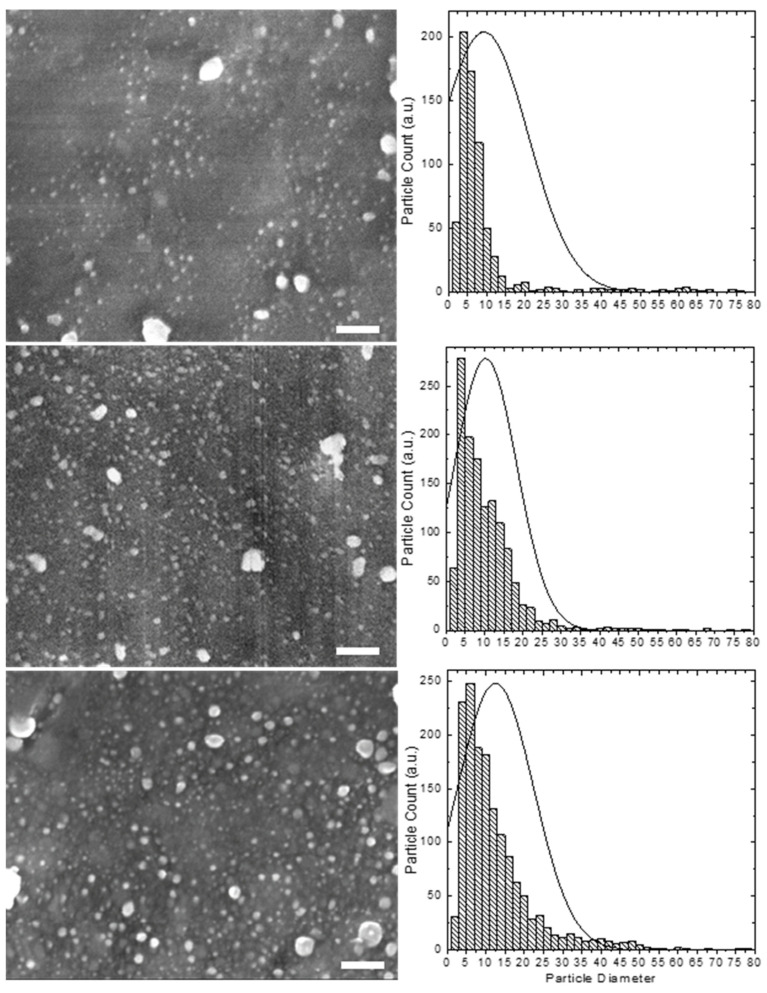
High-resolution SEM images (**left**) and corresponding particle size distribution histograms (**right**) of the Ag NPs embedded in resin beads prepared with increasing AgNO_3_ concentrations of 1 mM (**top**), 10 mM (**middle**) and 50 mM (**bottom**). Scale bar: 100 nm.

To further characterize the morphology and determine more accurately the size, distribution, and elemental composition of the embedded Ag nanoparticles in resin beads, we conducted SEM and EDS examinations. From Figure 3, it can be clearly seen that after the loading process, the resin beads maintained their micron-size spherical shape and integrity and did not show any significant sign of cracks or breakage, revealing high stability and most likely uniform distribution Ag nanoparticles throughout the resin. The homogenous surface and the presence of only very few aggregates or clusters on the resin surface indicate that the Ag nanoparticles are uniformly dispersed throughout the resin. The EDS spectra confirmed the purity and the presence of mainly metallic Ag NPs on the resin beads by the presence of two Ag peaks in the synthesized resin composites (Figure 3). Only minor amounts of Cl^−^ ions were observed in the loaded resin, indicating that most Cl^−^ ions were removed through the exchange process. The Ag NPs were also found to be evenly distributed throughout the surface of the resin beads and were buried and clearly visible as far as 10 μm from the bead surface, as confirmed by the EDS mapping. High-resolution SEM images and corresponding particle size distribution histograms of the resins coated with Ag NPs prepared with different AgNO_3_ concentrations are shown in Figure 4. These images clearly show that the Ag NPs are uniformly introduced and dispersed throughout the resin surface without any significant aggregation. A statistical analysis of the randomly dispersed Ag nanoparticles in these and other images yielded average particle sizes of 9.3 ± 11.5, 10.3 ± 8.1, and 12.5 ± 9.9 nm by increasing the AgNO_3_ concentrations from 1 to 10 to 50 mM, respectively (Figure 4). The resin samples prepared with high Ag content contained a higher population of Ag NPs; however, the observed particles were comparable in size and increased only slightly. Interestingly, the distribution curves appeared to be similar, with nearly most of the nanoparticles falling within the narrow range of 2–20 nm and only a few Ag NP clusters of 20–100 nm. A slightly greater number of larger particle sizes were observed for Ag-resin with higher impregnated Ag loading. Another interesting feature is that the size of nanoparticles seem to be comparable to the size of pores throughout the resin. The particle sizes are not so different from that of the XRD analysis agreeing well with the relatively rough estimate in terms of the Debye–Scherrer equation.

**Figure 5:**
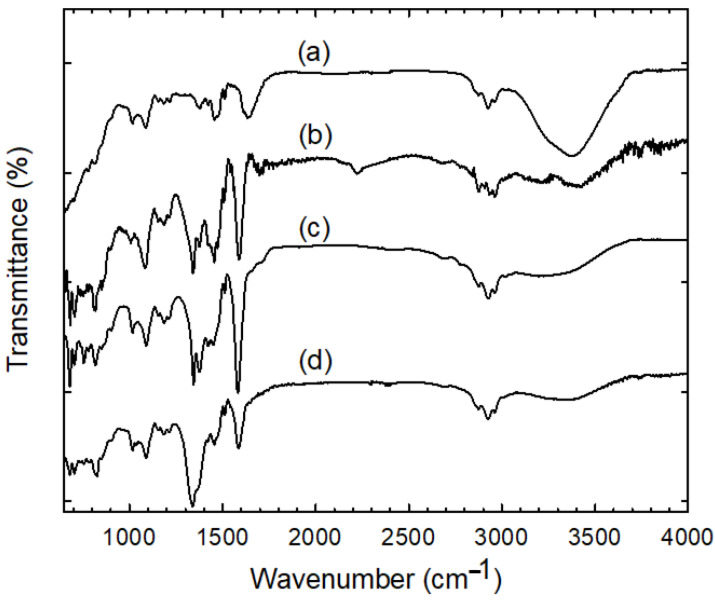
A comparison of FTIR spectra of the pure resin in chloride form (**a**) and those loaded with silver particles using increasing AgNO_3_ concentrations of 1 mM (**b**), 10 mM (**c**), and 50 mM (**d**).

Additional information about the functional groups and Ag sorption mechanism by anion exchange resin was obtained using FTIR analysis. Figure 5 shows a comparison of FTIR spectra of the pure resin in chloride form and those loaded with Ag particles using various AgNO_3_ concentration-containing solutions. Overall, all the spectra’s features were practically preserved; thus, one can conclude that no major compositional changes occur in the resin’s structure and functional groups after the exchange with BH_4_^−^ and subsequent retention of the Ag nanoparticles. The presence of Ag in the resin mostly manifested in the shifts in frequency and changes in the intensity of peaks assigned to the stretching and asymmetric vibrations of –N^+^(CH_3_)_3_ groups. The peak at 3432 cm^−1^ arising from the O–H stretching vibration due to the residual water adsorbed on the –N^+^(CH_3_)_3_ moiety was distinctly decreased and blue-shifted in the silver precursor-immobilized resin beads. The peaks at 1476 and 1454 cm^−1^ corresponding to C–H symmetric bending of the methyl groups of the quaternary ammonium was blue-shifted and strengthened [31]. Another notable difference was observed in the characteristic peak of C–N stretching vibration at 1340 and 1375 cm^−1^, which became significantly strong and sharp. The exchange with BH_4_^−^ and adsorption of Ag also disturbed the original C=C stretching vibrations of benzene rings, which were sharpened and blue-shifted to 1600 cm^−1^ with an intensity increase. The extra bands observed at 820, 755, and 680 cm^−1^ are likely associated with the C–H out-of-plane deformation vibrations of substituted benzene rings with contributions from the bending stretching vibrations in N–CH_3_. The rest of the peaks remained unchanged, which is characteristic of the polystyrene divinylbenzene matrix in the resin. The asymmetric and symmetric C-H stretching vibrations of CH_2_ and CH_3_ in the main chain were observed in the region of 2965–2873 cm^−1^. These peaks could also contribute from the methylene terminal group of the head group CH_3_–N^+^. The peaks in the region 1027–1180 cm^−1^ are likely related to the benzene ring’s C–H bonds in-plane deformation vibrations. Based on the above observations, it can be reasonably deduced that there is a strong interaction between the quaternary ammonium cationic moieties of the resin beads and the surface of Ag NPs, with the retention occurring mostly on these moieties.

**Figure 6:**
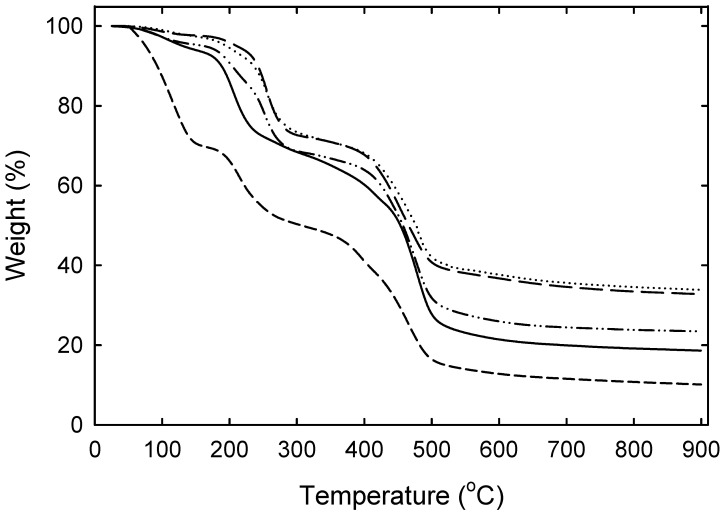
TGA curves of the pure resin in chloride form (----) and those loaded with Ag NPs using increasing AgNO_3_ concentrations of 1 mM (—), 10 mM (–••–) and 50 mM (— —), and 100 mM (•••).

Thermogravimetric analysis (TGA) was further carried out to provide quantitative results about the decomposition of the organic layer at high temperature and the loading weight ratio of Ag nanoparticles in the resin. Figure 6 compares the TGA curves of the unloaded resin beads and those loaded with Ag NPs using various AgNO_3_ concentration-containing solutions. The results show that all the samples possess three main weight loss peaks displaying a very similar pattern after pyrolyzing from room temperature to about 900 °C in a nitrogen flow. The first weight-loss step at low temperature below 180 °C is mainly due to evaporation of residual water, which is significant for the unloaded resin about 30% and quite small (less than 5%) for the loaded samples indicating the removal of absorbed water during the Ag loading. The second weight-loss step, which seems to occur in two steps between 180 to 280 °C and then to 350 °C, is due to the decomposition of pendant functional groups such as quaternary ammonium groups. At this stage, a lower onset weight loss rate was observed for loaded resins, which may be related to the complexing effect of these functional groups with Ag nanoparticles. The third degradation that took place at the temperature range between 350 to 500 °C is attributed to the polymer backbone decomposition. The pure resin produced a final residue mass fraction of about 10.2 wt%, consisting mainly of incombustible carbon ash. As expected, the presence of Ag further increased the weight content of residue after full degradation, and the effect is most marked for resin with higher Ag content. Assuming a constant resin residue after the TGA program is not possible to discard, the Ag contents estimated in the composite resin gradually increased from 8.5 to 9.4 to 13.3 to 22.7 to 23.7 wt% with increasing concentrations of the AgNO_3_ from 1 to 10 to 50 to 100 mM, respectively. The results also indicate that the resin is almost saturated with Ag nanoparticles when a concentration of 50 mM AgNO_3_ is used for loading. The Ag contents were consistent with those calculated from the synthesis before and after Ag loading in resin. In addition, the total content of silver on the saturated resin prepared with concentrations of 50 to 100 mM was further determined by the calibration curve ICP-OES. According to ICP-OES, the Ag contents were on the level of 21 and 23% ± 4%, which reasonably agree with those estimated by the TGA analyses.

The above results suggest that the nanoparticles are likely hosted in the nanoscale pores in the resin structure and are isolated. The resin has a highly developed structure of pores (with a size of about 2 to 100 nm) on the surface and within the framework. Thus, in principle, one might expect the formation of Ag particles in the same dimension as that of the pores, which serve as hosts in the resin structure. The concurrent effect of electrostatic stabilization by the charged ammonium functional groups and steric stabilization by the porosity seems to accommodate well with the growth of the nanoparticles toward their thermodynamically preferred sizes, but only up to a limit imposed by the rigid boundaries of polymer backbones in the resin [25]. These two effects could also explain the formation of a larger number of smaller particles of similar sizes at higher Ag loadings, as reflected in the particle size distributions.

The stability of the stored Ag NPs was also evaluated over time. Interestingly, the Ag nanoparticles were surprisingly stable in ambient air for more than four years after their preparation and unaffected, maintaining their colors, sizes, and dispersion according to X-ray diffraction and electron microscopy results. Thus it becomes more conclusive that the strong base functionalities and the nanoscale pores within the resin play the most significant stability role, as the former reveal strong affinity towards the metal surface through electrostatic interaction, and the latter isolates and limits the growth of the embedded Ag nanoparticles, preventing their aggregation and oxidation. The higher stability of these immobilized smaller AgNPs as compared to the colloidal AgNPs could represent an ideal design for practical long-term disinfection applications that can be used multiple times with good efficacy use reducing the toxic effects on consumption of this disinfected water due to less carry-over of free AgNPs into the aqueous medium.

**Figure 7:**
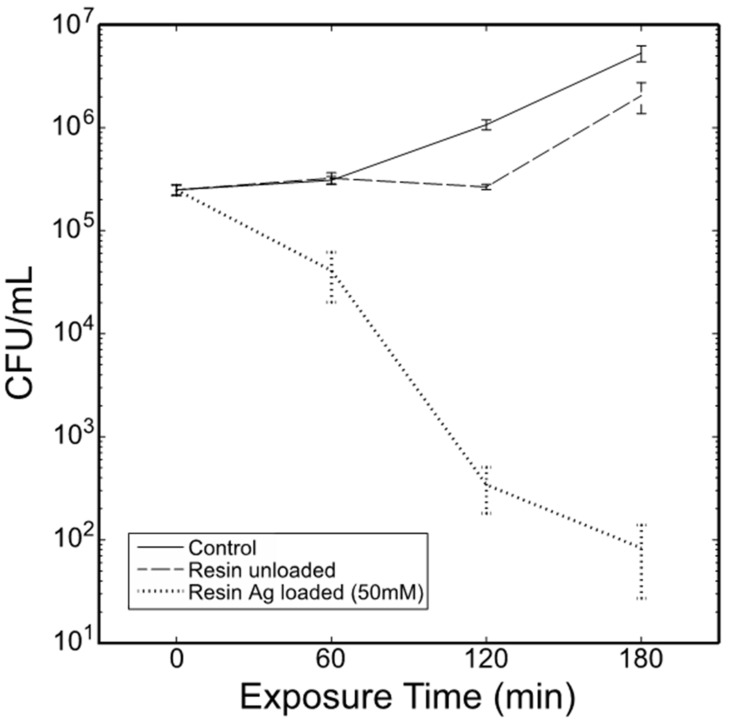
Comparative in vitro antibacterial activity of a single unloaded resin bead and a single resin bead loaded with 22.7 wt% Ag nanoparticles (made with 50 mM AgNO_3_) against *Escherichia coli*.

To elucidate their disinfection and antimicrobial ability, the Ag-loaded resins made with 22.7% (*w*/*w*) Ag nanoparticles were tested for antibacterial activity in vitro against *E. coli* as a model microbial contaminant in water. In a typical experiment, aliquots of *E. coli* B cells (~62,000 CFU) were added to wells of a 96-well-microtiter plate containing only cells, cells with a single unloaded resin bead or cells with a single Ag loaded resin bead and incubated for 3 h at 37 °C with agitation (Figure 7). Viable counts of each growth condition at each time point revealed significant effects of growth conditions, exposure time, and interaction of growth conditions and exposure time (Table 1). Control cells lagged for approximately 60 min but entered the early log phase by the 3-h sampling. Interestingly, unloaded resin beads caused cells to lag for an additional 60 min before entering the log phase. Ag embedded in resin beads showed a significantly reduced number of colony-forming units (CFU) at 60 min (Table 2). This trend continued until direct plating of cells (100-μL aliquots) reached an approximate mean of 80 CFU/mL by the 3-h sampling. The results show greater than 99% antibacterial activity against *E. coli* using only a single Ag loaded resin bead, demonstrating greater efficacy than colloidal Ag NPs and AgCl substrates that release Ag ions [32,33,34,35]. According to ICP, the maximum amount of silver released from the Ag/resin beads even after 3 h shaking in nutrient broth was about 0.2% of the total amount of silver immobilized in the resin beads. The resin bead design allows the realization of an effective and practical disinfection system at a lower Ag concentration. In addition, the higher stability of these immobilized Ag NPs also avoids the excess release of free Ag NPs into an aqueous medium and prevents their oxidation or aggregation over time, allowing long-term antimicrobial activity, repeated use, and reduction of the associated environmental threats.

Based on these preliminary results, these immobilized AgNPs may mechanistically act through direct contact behavior and/or slow release of Ag^+^ ions, which is in concurrence with previous studies that used Ag NPs in colloidal form and those embedded in different substrates [32,33,34,35,36]. Several reports have shown that AgNPs, when in direct contact with bacterial cells, stimulate the formation of irregular pits in the cell membrane through interaction with proteins and nucleic acids, which gradually damage the integrity of the cell wall and cause the leakage of cellular components [33,34,35]. Similarly, silver ions released from immobilized Ag NPs, in particular when placed in contact with the cell surface, can easily attach to the cell walls and enter into the cytoplasm through interaction with thiolates (of the cell membranes) or phosphates (in DNA), thereby resulting in several cellular dysfunctions such as rupturing the cell wall, protein denaturation, and blocking cell respiration [33,34,35,36,37,38]. For example, Ag NP firmly grafted to the adhesive mercaptopropyltrimethoxy silane (MPTS) or polyethylenimine (PEI) layer NP-coated slides showed an efficient and long-lasting effect against *E. coli.* In both cases, the microbicidal effect was attributed to the slow and sustained release of Ag^+^ from the NP surface [37]. In other reports, the attachment of Ag^+^—ligand complexes to a surface has also been found in most cases to significantly slow down the Ag^+^ release, resulting in an efficient antibacterial activity [38]. Further studies on multiple bacterial species and strains are needed to confirm these hypotheses and better elucidate the bactericidal action of these immobilized AgNPs and how they influence the development, metabolism, and bacterial composition.

## 4. Summary

In summary, Ag NPs embedded in anion exchange resin have been successfully prepared through a facile solution-phase synthesis method by direct chemical reduction of Ag^+^ ions with BH_4_^−^ exchange resin. The process overcomes the inherent difficulties of using complex precursors for ion exchange or pre-fabricated nanoparticles for storing within resin, causing agglomeration and inhomogeneous distribution. The results confirmed the presence of crystalline Ag NPs incorporated into the resin beads with diameters on the order of 10 nm and only a few Ag NP clusters of 20–100 nm that are homogeneously distributed throughout the resin. The loading amount of Ag NPs in resin composites gradually increased by increasing the AgNO_3_ concentration, while their sizes remained roughly comparable. A slightly greater number of larger particle sizes were observed for Ag-resin with higher impregnated Ag loading. Meanwhile, the nanocomposites exhibited high chemical stability and reusability. These nanocomposites showed nearly 100% antibacterial activity against *Escherichia coli*, allowing the realization of an effective disinfection system while reducing the associated environmental threats of Ag release. The same coating method may be used to design and prepare other metal nanoparticles with satisfactory performance and eco-friendly advantages for efficient and promising applications. While we limited our study to the antibacterial application, the current method can potentially be exploited for other interesting purposes, such as catalysis, filtration systems, SERS-based diagnostics, and sensing.

## Figures and Tables

**Table 1:** Two-way ANOVA of effects of cell treatment (no bead, unloaded resin bead, and the resin bead loaded with 22.7% (*w*/*w*) Ag nanoparticles) and exposure time on the growth of *E. coli* B over three hours. Source.

Source	df ^1^	SS ^2^	MS ^3^	*F* ^4^	*p* ^5^
Cell treatment	2	2.24 × 10^13^	1.12 × 10^13^	24.96	**6.45 × 10^−6^**
Exposure time	2	2.70 × 10^13^	1.35 × 10^13^	30.2	**1.77 × 10^−6^**
Treatment × exposure time interaction	4	2.25 × 10^13^	5.63 × 10^12^	12.57	**4.70 × 10^−5^**
Error	18	8.06 × 10^12^	4.48 × 10^11^		
Total	26	8.00 × 10^13^			

^1^ Degrees of freedom. ^2^ Sum of squares. ^3^ Mean squared. ^4^ *F*-statistic calculated for each term. ^5^ Bolded *p*-values indicate statistical significance.

**Table 2:** Pairwise *t*-tests between time points within each growth condition.

Pairwise Comparison		df ^1^	*t* ^2^	*p* ^3,4^
Control—60 min	Unloaded resin—60 min	4	0.3024	0.7774
Control—60 min	Ag loaded resin—60 min	4	−8.0398	**0.0013**
Unloaded resin—60 min	Ag loaded resin—60 min	4	−6.1445	**0.0036**
Control—120 min	Unloaded resin—120 min	4	−6.8356	**0.0024**
Control—120 min	Ag loaded resin—120 min	4	−9.1848	**0.0008**
Unloaded resin—120 min	Ag loaded resin—120 min	4	−17.5066	**0.0001**
Control—180 min	Unloaded resin—180 min	4	−2.8244	0.0476
Control—180 min	Ag loaded resin—180 min	4	−5.704	**0.0047**
Unloaded resin—180 min	Ag loaded resin—180 min	4	−3.0061	0.0397

^1^ Degrees of freedom. ^2^ *t*-statistic for pairwise comparison. ^3^ Significance levels Bonferroni-adjusted for multiple comparisons (α = 0.05/9 = 0.0056). ^4^ Bolded *p*-values indicate statistical significance.

## Data Availability

Not applicable.
